# Overexpression of Semaphorin-3E enhances pancreatic cancer cell growth and associates with poor patient survival

**DOI:** 10.18632/oncotarget.13704

**Published:** 2016-11-29

**Authors:** Lin-Kin Yong, Syeling Lai, Zhengdong Liang, Ethan Poteet, Fengju Chen, George van Buren, William Fisher, Qianxing Mo, Changyi Chen, Qizhi Yao

**Affiliations:** ^1^ Michael E. DeBakey Department of Surgery, Division of Surgical Research, Baylor College of Medicine, Houston, TX, USA; ^2^ Interdepartmental Program in Translational Biology and Molecular Medicine, Baylor College of Medicine, Houston, TX, USA; ^3^ Department of Pathology, Michael E. DeBakey VA Medical Center and Baylor College of Medicine, Houston, TX, USA; ^4^ Duncan Cancer Center, Baylor College of Medicine, Houston, TX, USA; ^5^ Department of Medicine, Baylor College of Medicine, Houston, TX, USA; ^6^ Michael E. DeBakey Department of Surgery, Division of General Surgery, Baylor College of Medicine, Houston, TX, USA; ^7^ Center for Translational Research on Inflammatory Diseases (CTRID), Michael E. DeBakey VA Medical Center, Houston, TX, USA

**Keywords:** semaphorin 3E, pancreatic cancer

## Abstract

Semaphorin-3E (Sema3E) is a member of an axon guidance gene family, and has recently been reported to contribute to tumor progression and metastasis. However, its role in pancreatic cancer is yet unknown and uncharacterized. In this study, we showed that Sema3E is overexpressed in human pancreatic cancer, and that high Sema3E levels are associated with tumor progression and poor survival. Interestingly, we also observed Sema3E expression in the nucleus, even though Sema3E is reported to be a secreted protein. Overexpression of Sema3E in pancreatic cancer cells promoted cell proliferation and migration *in vitro*, and increased tumor incidence and growth *in vivo*. Conversely, knockout of Sema3E suppressed cancer cell proliferation and migration *in vitro*, and reduced tumor incidence and size *in vivo*. Moreover, Sema3E induced cell proliferation via acting through the MAPK/ERK pathway. Collectively, these results reveal an undiscovered role of Sema3E in promoting pancreatic cancer pathogenesis, suggesting that Sema3E may be a suitable prognostic marker and therapeutic target for pancreatic cancer.

## INTRODUCTION

Pancreatic cancer is the third leading cause of cancer death in the United States, with a 5-year survival rate of approximately 7% [1, 2]. The majority of pancreatic cancers are pancreatic adenocarcinomas (PDAC) (90%), while there is a lower incidence for neuroendocrine tumors (5%). PDAC is usually diagnosed only at the late stages due to the disease's asymptomatic nature, a lack of sensitive markers, and challenges in imaging early-stage tumors. PDAC cells are also highly resistant to conventional treatments, including chemotherapy, radiotherapy, and molecularly targeted therapies. Moreover, the genetic complexity of the cancer poses a major challenge in developing targeted therapies. The dense stroma infiltrating the tumor also supports tumor growth and is a physical barrier to the penetrance of chemotherapy drugs [3]. Therefore, two outstanding challenges exist: the need for better detection methods, and the need for more effective and targeted treatments. A clear understanding of the molecular mechanisms of PDAC pathogenesis will help in identifying key prognostic and therapeutic targets.

The axon guidance molecule, Semaphorin-3E (Sema3E), has been identified in the past several years to be overexpressed in breast, colon, and ovarian cancers, and has also been shown to be associated with worse disease progression or metastatic cancer. [4–8]. Genomic analyses of large cohorts of human PDAC samples have uncovered genetic aberrations in axon guidance genes, specifically *SEMA3E* gene which was found to be aberrantly amplified (copy number variations, CNV) in the majority of the samples [9–11]; Therefore, the significance of amplified Sema3E gene in pancreatic cancer warrants further study. Biankin et al. [9] also found that Sema3E mRNA levels were elevated in the genetically engineered spontaneous pancreatic cancer mouse model (LSL-Kras^G12D/+^; LSL-Trp53^R172H/+^; Pdx-1-Cre) [12]. Sema3E, amongst other Sema3 proteins, has also been found to be overexpressed in a study of human PDAC samples, but no correlation with clinical parameters could be established [13]. Thus, there is a need for further study of the effects and significance of Sema3E's aberrant expression in PDAC.

Sema3E is a class 3 secreted glycoprotein encoded by the *SEMA3E* gene. The full length protein of Sema3E is 775 amino acids (a-a) long and has a molecular weight of 87 kDa. Furin and furin-like convertases can cleave the protein at the 560 a-a position to yield a biologically active 61 kDa fragment and a 25 kDa c-terminal fragment [5]. Unlike other class 3 semaphorins which require binding to a neuropilin co-receptor, Sema3E can bind directly to its primary receptor Plexin D1 to activate cell signaling, or can bind a heterodimer complex of Plexin D1 and Neuropilin-1 [14, 15]. The 61 kDa fragment, like the full-length protein, can bind directly to Plexin D1 to activate cell signaling; such binding has been shown to contribute to cancer cell invasiveness and metastatic spreading by transactivating erythroblastic leukemia viral oncogene homolog 2 (ErbB2) or epidermal growth factor receptor (EGFR) oncogenic tyrosine kinase receptors in colon cancer cells [6] or by inducing nuclear localization of zinc finger protein Snail1 in ovarian cancer cells [7].

In this study, we found that Sema3E was significantly overexpressed in human pancreatic cancer, and high levels of the protein correlated with worse survival. Notably, given that Sema3E is known to be a secreted protein, we made the striking observation of Sema3E's expression in the nucleus, in addition to its localization in the cytoplasm. Overexpression of wildtype Sema3E in human pancreatic cancer cell lines enhanced cell growth and migration in cell culture conditions, while knockout of Sema3E reduced cell proliferation and migration. In an orthotopic mouse xenograft model, Sema3E-overexpressed cells exhibited greater tumor incidence and growth, whereas Sema3E-knockout cells had reduced tumor incidence and growth. These data collectively suggest that aberrant amplification of Sema3E gene contributes to pancreatic cancer pathogenesis and could be a suitable prognostic marker and therapeutic target for pancreatic cancer.

## RESULTS

### Sema3E is overexpressed in human pancreatic cancer

**Figure 1:**
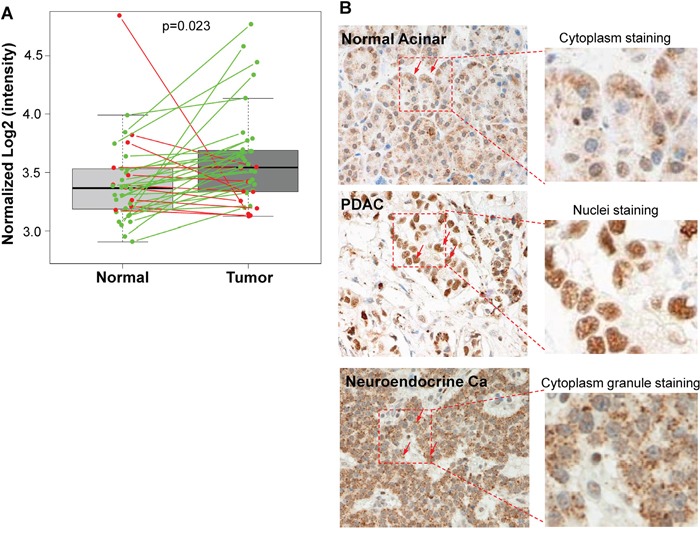
Sema3E is overexpressed in human pancreatic adenocarcinoma (PDAC) **A**. Paired analysis of Sema3E gene expression in the GEO PDAC microarray dataset GDS4103 (n=72) shows a significant increase in Sema3E gene expression in tumors compared to matched normal tissue controls (Student's *t-test*, *p*=0.02). The green lines indicate paired samples with upregulated Sema3E expression, while red lines indicate paired samples with downregulated Sema3E expression. **B**. Representative IHC staining for Sema3E in human tissues: acinar cells of normal pancreas (left panel), pancreatic adenocarcinoma (PDAC) (middle panel), and pancreatic neuroendocrine carcinoma (right panel). Arrows point to the location of Sema3E staining in brown in each of these tissues: mostly cytoplasmic in normal acinar cells, mostly nuclear in PDAC, and in cytoplasmic granules in neuroendocrine carcinoma. Red squares are small sections that got further magnified to shown clear cellular localization of Sema3E staining.

To assess the clinical significance of Sema3E in pancreatic cancer, we sought to determine Sema3E expression in samples obtained from different sources. The first source of sample data was obtained from the publicly available GEO dataset, GDS4103, which consisted of 78 human PDAC and matched normal control samples. As shown in Figure 1A, there was a statistically significant higher expression of Sema3E transcription in a paired analysis of tumor samples vs. matched controls (*p*=0.023), indicating that Sema3E is overexpressed in PDAC. Another source was from a cohort of patient samples in the Michael E. DeBakey VA Medical Center, where the type and number of samples are outlined in Table 1. Sema3E-specific Immunohistochemical staining (IHC) was performed on a tissue array, and as shown in Figure 1B, there was a higher intensity of Sema3E staining in ductal tumor cells (middle panel) compared to normal pancreatic acinar cells (left panel). Interestingly, we observed that Sema3E was strongly expressed in the nuclei of cancer cells while its expression in normal cells was weak and mostly confined to the cytoplasm. In contrast, Sema3E was strongly expressed in the cytoplasmic granules of neuroendocrine carcinoma tumor cells (right panel). Sema3E expression levels were determined to be higher in tumors vs. normal controls in both sources of patient samples, indicating that Sema3E may be involved in pancreatic cancer pathogenesis.

**Table 1:** Summary of human pancreas samples from VA medical center used in immunohistochemical analysis of Sema3E expression

	Type of sample	# of cases(% of total)	Total # of cases (% of grand total)
**Neoplasm**			**44**
	*Malignant*		*33 (75%)*
	Adenocarcinoma	25 (76%)	
	Sarcomatoid carcinoma	1 (3%)	
	Neuroendocrine carcinoma	7 (21%)	
	*Uncertain*		*8 (18%)*
	Intraductal papillary mucinous neoplasm	2 (25%)	
	Neuroendocrine tumor	6 (75%)	
	*Benign*		*3 (7%)*
	Pancreatic intraepithelial neoplasm	1 (33%)	
	Serous microcystic adenoma	2 (67%)	
**Non-neoplasm**			**5**
	Fibrosis	1 (20%)	
	Acute pancreatitis	1 (20%)	
	Islet hyperplasia	2 (40%)	
	Lymphoepithelial cyst	1 (20%)	
**Normal**			**5**
	Associated adenocarcinoma	2 (40%)	
	Associated neuroendocrine carcinoma	1 (20%)	
	Associated neuroendocrine tumor	2 (40%)	

### High Sema3E expression is associated with worse disease progression and poor survival

**Figure 2:**
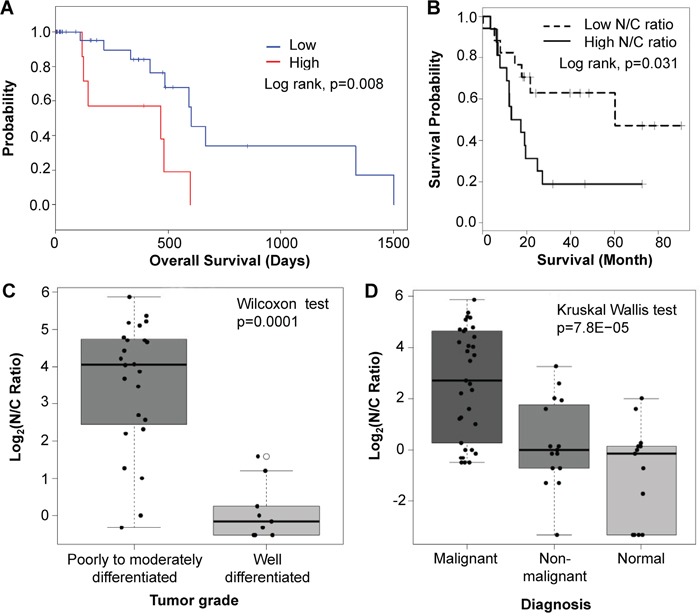
High Sema3E expression is associated with poor survival, tumor progression, and more aggressive disease **A**. Kaplan-Meier survival analysis of PDAC samples from the TCGA PAAD RNAseq exon expression dataset, stratified by high (top 25%) and low (bottom 75%) Sema3E gene expression, shows that high Sema3E expression is correlated with poor survival (Log-rank test, p=0.008). **B**. Likewise, Kaplan-Meier survival analysis of samples from the VA medical center dataset, stratified by high and low N/C ratio of Sema3E expression, shows that high N/C ratio of Sema3E expression correlates with poor survival (Log-rank test, *p*=0.03). **C**. Statistical analysis of samples from the VA Medical Center dataset shows that poorly to moderately-differentiated tumors have a significantly higher N/C ratio of Sema3E expression compared to well-differentiated tumors (Wilcoxon signed-rank test, *p*=0.0001). **D**. Statistical analysis of the same VA Medical Center dataset shows that high N/C ratio of Sema3E expression is associated with more aggressive pancreatic cancer subtypes, since adenocarcinomas, the most aggressive form of pancreatic cancer, express the highest N/C ratios, followed by the less aggressive neuroendocrine carcinoma, then followed by normal pancreatic tissue controls (Kruskal-Wallis test, *p*=8e-05).

To determine if high expression of Sema3E is indicative of a poor prognosis, we calculated a Kaplan-Meier survival analysis of the TCGA PAAD RNAseq exon expression data (Figure 2A), which indicated that samples expressing high levels of Sema3E RNA had significantly poorer survival than samples expressing low levels of the protein (*p*=0.008). As mentioned earlier, in the VA Medical Center patient samples, we observed strikingly strong staining of Sema3E in the nuclei of tumor cells compared to mostly cytoplasmic staining in healthy cells. We therefore computed a nuclear-to-cytoplasm ratio (N/C ratio) of Sema3E expression, which is the nuclear to cytoplasm staining intensity of Sema3E in a given sample. Kaplan-Meier survival analysis of samples stratified by high and low N/C expression ratios (Figure 2B) revealed that patients with a high N/C ratio had significantly poorer survival compared to patients with a low N/C ratio (*p*=0.031), reflecting the importance of both cellular localization and expression level of Sema3E in influencing disease progression. Indeed, as shown by Figure 2C, poorly to moderately differentiated samples had significantly higher N/C ratios compared to well-differentiated samples (*p*=0.0001). A high N/C ratio was also associated with more aggressive forms of the cancer (Figure 2D), since highly malignant PDAC samples had the highest N/C ratio, followed by the less malignant neuroendocrine pancreatic cancer, and finally normal tissue, which had the lowest N/C ratio of the analyzed samples (*p*=7.8e-05). Altogether, these data indicate that Sema3E may play an adverse role in the progression of human pancreatic cancer.

### Sema3E is overexpressed in human pancreatic cancer cell lines

**Figure 3:**
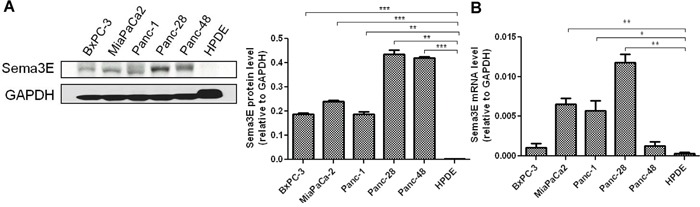
Sema3E is overexpressed in human PDAC lines A panel of human PDAC cell lines, plus the normal pancreatic ductal cell line, HPDE, was screened for Sema3E protein and gene expression. **A**. Immunoblot analysis (left panel) show that all of the PDAC cell lines express moderate to high levels of Sema3E protein, while HPDE expressed very minimal levels of the protein. Densitometry analysis of Sema3E protein levels relative to GAPDH protein levels are shown on the right panel. (**p<0.01; ***p<0.001). **B**. RT-PCR analysis in the same panel of PDAC cell lines confirms low gene expression of Sema3E in HPDE as compared to the cell lines, MiaPaCa-2, Panc-1 and Panc-28. (*p<0.05; **p<0.01). All data are represented as mean ± S.E.M, and are representative of at least 3 independent experiments.

We also sought to determine if Sema3E was overexpressed in human PDAC cell lines by analyzing the levels of Sema3E protein and mRNA in a panel of cell lines generated from primary human PDAC. As shown in Figure 3A, immunoblot analysis of Sema3E protein expression in five PDAC cell lines as well as the transformed human pancreatic ductal epithelium (HPDE) cells serving as a non-tumor control cell line, revealed moderate to high expression of the 87 kDa full-length form of Sema3E protein in all five cancer cell lines, but not in the HPDE control cells. RT-PCR analysis of Sema3E mRNA levels, as shown in Figure 3B, likewise showed that HPDE cells contain low levels of Sema3E mRNA compared to the PDAC cell lines, and this is statistically significant for MiaPaCa-2, Panc-1, and Panc-28 cell lines.

### Sema3E is observed in both the cytoplasm and nucleus of Sema3E-overexpressing stable cell lines

To determine the effects of Sema3E on the functions of pancreatic cancer cells, we generated stable Sema3E-overexpressing and corresponding vector control cell lines via transduction of MiaPaCa-2 and Panc-48 cells with a lentiviral vector coding for Sema3E cDNA (-S3E) or vector control (-VCtrl). As shown in Supplementary Figure S1A, Sema3E-overexpressing Mia-S3E and Panc48-S3E cells had higher cytoplasmic expression of both the full-length 87 kDa and the truncated 61 kDa forms of Sema3E compared to their respective controls (Mia-VCtrl and Panc48-VCtrl). Sema3E was also expressed in the nuclear fractions, corroborating the observations we have made about Sema3E localization in the nucleus in patient PDAC samples. However, unlike in the cytoplasmic fractions, only the full-length protein was increased in the nuclear fractions. In the culture supernatant, both the full length and 61 kDa isoforms of the protein were found in significant amounts in the Sema3E-overexpressing cells but not in the controls. RT-PCR analysis of Sema3E mRNA levels in these cells, confirmed higher levels of Sema3E mRNA in the Sema3E-overexpressing cells compared to the controls (Supplementary Figure S1B).

Immunofluorescence staining analysis of the Sema3E-overexpressing cells, as shown in Supplementary Figure S2, indicates a significant increase in intensity of Sema3E staining in both Mia-S3E and Panc48-S3E cells as compared to their respective controls. Sema3E staining could be observed in both the nucleus and cytoplasm of both control cell lines, and in the Sema3E-overexpressing cells, the levels of Sema3E protein in both cellular compartments were increased, corroborating the results obtained from immunoblot analyses.

### Sema3E overexpression increases cell growth, clonogenic potential, and migration

**Figure 4:**
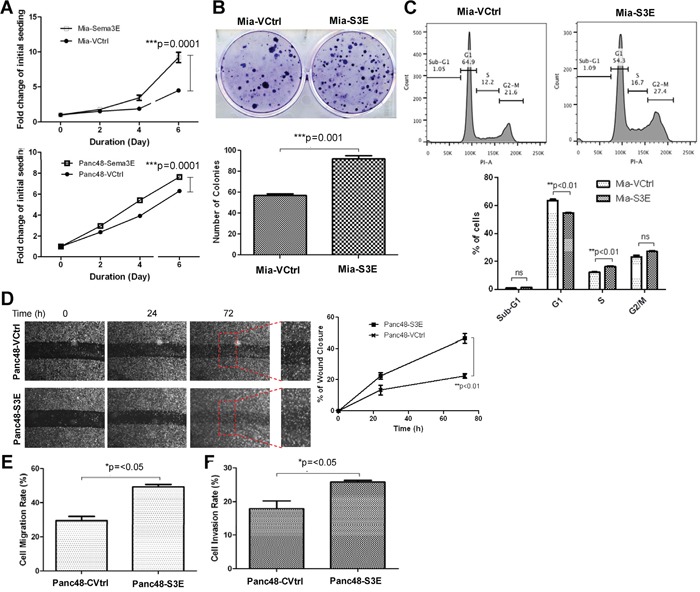
Overexpression of Sema3E in PDAC cell lines increases cell growth, proliferation and clonogenic potential, as well as cell migration **A**. MTT assay shows that both Mia-S3E and Panc48-S3E cells had significantly greater cell growth over the period of 6 days compared to their respective controls (two-way ANOVA and Bonferroni posttest, ***p<0.001). **B**. In a colony formation assay, Mia-S3E cells had a higher rate of colony growth compared to the control, indicating increased clonogenic potential (Student's *t-test*, ***p<0.001). **C**. Cell cycle analysis shows an increased rate in cell proliferation in Mia-S3E cells compared to the control, as indicated by a significant G1 to S phase shift (Student's *t-test*, **p<0.01). **D**. A wound healing scratch assay shows that Panc48-S3E cells had increased cell movement towards the center of the wound scratch compared to the vector control over 72 h (two-way ANOVA and Bonferroni posttest,**p<0.01). Red squares are small sections in the wound area that got further magnified to demonstrate more wound closure in Panc48-S3E cells vs. the control cells Panc48-VCtrl. **E**. In a transwell migration assay using uncoated transwells, Sema3E-overexpressing Panc-48 cells similarly had increased cell migration compared to the control (Student's *t-test*, *p<0.05). **F**. In a transwell invasion assay using transwells coated with matrigel, Sema3E-overexpressing Panc-48 cells had increased cell invasion compared to the control (Student's *t-test*, *p<0.05). All data are represented as mean ± S.E.M, and are representative of at least 3 independent experiments.

To determine the effects of Sema3E overexpression on cell growth, an MTT (3-(4,5-dimethylthiazol-2-yl)-2,5-diphenyltetrazolium bromide) assay was performed. As shown in Figure 4A, Sema3E-overexpressing MiaPaCa-2 and Panc-48 cells had significantly higher cell proliferation over a period of 6 days compared to the respective vector controls, as measured by the fold-change of absorbance at the indicated time points with respect to absorbance at day 0.

To determine the effects of Sema3E overexpression on clonogenic potential, a colony formation assay was performed. As shown in Figure 4B, Mia-S3E cells formed more colonies than the vector control, Mia-VCtrl, over a period of 14 days, indicating that Sema3E overexpression confers greater clonogenic potential.

To further examine the effects of Sema3E overexpression on increasing cell growth, cell cycle analysis was performed using propidium iodide (PI). As indicated by Figure 4C, Mia-S3E cells had a greater number of cells in the S phase compared to the control, as well as a corresponding fewer number of cells in the G1 phase, indicating that Sema3E overexpression induced more G1 to S phase transition, implying that Sema3E could drive cell proliferation.

To determine the effects of Sema3E overexpression on cell migration, a wound healing assay was performed. As shown in Figure 4D, more cells migrated into the wound area for the Sema3E-overexpressing Panc-48 cells over a period of 72 h as compared to the control. A quantification of images taken of the wound area also showed that Panc48-S3E cells had a significantly higher rate of wound closure compared to the control. In addition, a Boyden chamber assay was performed using transwells to determine the rate of cell migration or invasion from the top of the membrane in the chamber to the bottom of the membrane. Uncoated transwells were used to assess cell migration and transwells coated with matrigel (to simulate the extracellular matrix) were used to assess cell invasion. As shown in Figure 4E and 4F, Panc48-S3E cells had significantly higher rates of cell migration and invasion compared to the control.

### Knockout of Sema3E in a pancreatic cancer cell line decreases cell growth and migration

**Figure 5:**
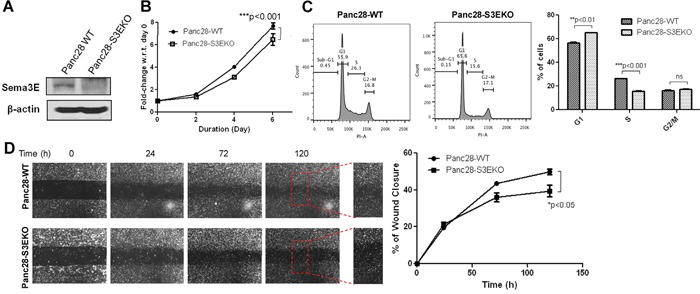
Knockout of Sema3E in a PDAC cell line decreases cell growth and proliferation, as well as cell migration Sema3E-knockout PDAC cells were generated with Panc-28 cells using the CRISPR-Cas9 system, with sgRNA targeting *SEMA3E* gene. **A**. Immunoblot analysis shows the absence of the 87 kDa full-length protein in Panc28 Sema3E-knockout (Panc28-S3EKO) cells, compared to the parental wild type cells (Panc28-WT), confirming successful knockout of Sema3E protein. **B**. MTT assay shows that Panc28-S3EKO cells had significantly reduced cell growth over the period of 6 days compared to the WT control (two-way *ANOVA* and Bonferroni posttest, ***p<0.001). **C**. Cell cycle analysis shows a decreased rate in cell proliferation in Panc28-S3EKO cells compared to the control, as indicated by a significant S to G1 phase shift (Student's *t-test*, **p<0.01). **D**. A wound healing scratch assay shows that the Sema3E-knockout cells had less cell movement towards the center of the wound scratch, compared to the parental wild type control (two-way ANOVA and Bonferroni posttest,*p<0.05). Red squares are small sections in the wound area that got further magnified to demonstrate less wound closure in Panc28-S3EKO cells vs. the control cells Panc28-WT. All data are represented as mean ± S.E.M, and are representative of at least 3 independent experiments.

Having established the effects of Sema3E gain-of-function in PDAC cells, we sought to investigate the effects of Sema3E loss-of-function in PDAC cells. Panc-28 cells were found to have the highest protein and mRNA expression levels of Sema3E via immunoblot and RT-PCR analyses, and therefore were selected for the generation of a Sema3E knockout cell line. Sema3E-knockout Panc-28 stable cells, Panc28-S3EKO, were generated using the CRISPR-Cas9 gene editing system. Sema3E was successfully knocked out in Panc28-S3EKO cells, as indicated by immunoblot analysis (Figure 5A). DNA sequencing of these cells showed that Sema3E exon 1 was knocked out via indel mutation 83648437-38 and 83648445-50 (data not shown).

To determine the effects of Sema3E knockout on cell growth, an MTT assay was performed. Figure 5B shows that by the end of day 6, Panc28-S3EKO cells had a significantly lower rate of cell growth compared to the wild-type parental (WT) control cells. Cell cycle analysis using PI (Figure 5C) shows that Sema3E-knockout cells had fewer cells in the S phase as well as more resting cells in the G0/G1 phase compared to the control, indicating that fewer cells were undergoing G1 to S phase transition after loss of Sema 3E. Together, these results indicate that knockout of Sema3E decreases cell growth via suppressing cell proliferation.

To determine the effects of Sema3E knockout on cell migration, a wound healing assay was performed. As shown in Figure 5D, by the end of 120 h (5 days), Sema3E-knockout Panc-28 cells had a lower rate of wound closure compared to the parental WT cells, indicating that loss of Sema3E suppressed cell migration.

### Overexpression of Sema3E enhances tumor growth *in vivo*

**Figure 6:**
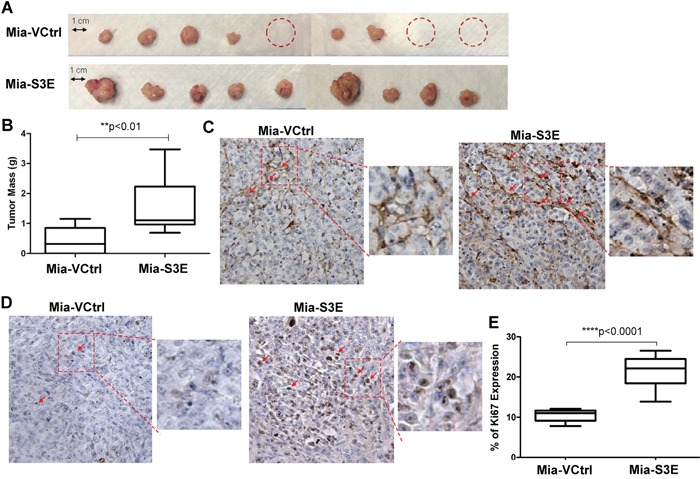
Overexpression of Sema3E increases cancer cell proliferation and tumor growth in vivo Sema3E-overexpressing Mia-S3E cells as well as the control cells were orthotopically implanted into the pancreas of 6 week old nude mice (n=9 per group). Mice were sacrificed at 8 weeks post-implantation, and tumors were explanted and analyzed. **A**. Images of the tumors show that overall, mice injected with Mia-S3E cells had greater incidence of tumor formation as well as larger tumors compared to mice injected with the control Mia-VCtrl cells. Mia-S3E tumors also appeared more vascularized than the control tumors. Red circles indicate no tumor incidence in mice. **B**. Tumors in mice inoculated with Mia-S3E cells had greater mass than tumors in mice inoculated with Mia-VCtrl cells (Student's *t-test*, **p<0.01). **C**. Expression of CD31 (brown staining), a marker of endothelial cells, in representative tumor tissues from Mia-VCtrl and Mia-S3E groups. Greater CD31 expression in Mia-S3E tumors compared to Mia-VCtrl is indicative of the presence of more microvessels in the Sema3E-overexpressing tumors. **D**. Expression of Ki67 (brown staining), a marker of cell proliferation, in representative tumor tissues from Mia-VCtrl and Mia-S3E groups. Ki67 expression was greater and stronger in tumors from Mia-S3E group compared to the control group. **E**. The extent of Ki67 expression in tumor tissues was quantified using the ImmunoRatio application plugged into ImageJ; there was a significantly higher Ki67 expression in tumors from the Mia-S3E group compared to tumors from the Mia-VCtrl group (Student's *t-test*, ****p<0.0001). Red squares are small sections that are further magnified for a clear demonstration of positive staining. All data are represented as mean ± S.E.M.

To investigate the effects of Sema3E overexpression *in vivo*, we implanted Sema3E-overexpressing MiaPaCa-2 (Mia-S3E) and the corresponding control cells (Mia-VCtrl) orthotopically into the pancreas of nude mice. As shown in Figure 6A, there was a higher incidence of tumor formation for mice inoculated with Mia-S3E cells compared to mice inoculated with Mia-VCtrl cells (100% and 66.7% respectively). In the same figure, the average tumor size was larger in the Mia-S3E group than in the control group. Figure 6B shows that tumors in the Mia-S3E group had significantly greater mass compared to tumors in Mia-VCtrl group. From Figure 6A, we also observed that Mia-S3E tumors appeared to be more vascularized than Mia-VCtrl tumors, so we performed an IHC staining for CD31, a widely used marker for endothelial cells. As shown in Figure 6C, Mia-S3E tumors had a greater abundance of microvessels compared to Mia-VCtrl tumors, as indicated by CD31 staining.

Ki-67 staining of the tumor sections (Figure 6D) showed that tumors in Mia-S3E group had higher Ki-67 expression levels compared to tumors in Mia-VCtrl group (Figure 6E), indicating that there was greater cell proliferation in Sema3E-overexpressing tumors compared to the controls, which could explain why Mia-S3E tumors had greater overall tumor mass. Altogether, these results show that overexpression of Sema3E enhanced tumor growth in an orthotopic *in vivo* setting.

### Knockout of Sema3E decreases tumor growth *in vivo*

**Figure 7:**
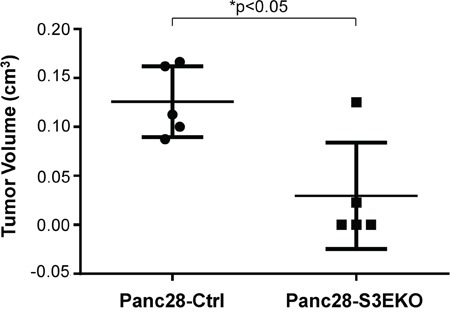
Knockout of Sema3E reduces tumor incidence and tumor growth in vivo Sema3E-knockout Panc28-S3EKO cells as well as the corresponding control Panc28-Ctrl cells (2 × 10^6^ cells) were orthotopically implanted into the pancreas of 6 week old nude mice (n=5 per group). Mice were sacrificed at 8 weeks post-implantation, and pancreas were explanted and analyzed. Mice injected with Panc28-S3EKO had reduced tumor incidence (only 2 out of 5 animals showed tumor growth) compared to the control (Panc28-Ctrl) (all 5 animals had tumor growth). Extracted tumors from the pancreas of these mice were measured, and tumor volume was calculated from measurements of length and width. Tumors from the Panc28-S3EKO group had significantly lower tumor volume than tumors from the Panc28-Ctrl group (Student's *t-test*, *p<0.05). All data are represented as mean ± S.E.M.

To investigate the effects of Sema3E knockout *in vivo*, we implanted Sema3E-knockout Panc-28 cells (Panc28-S3EKO) and the corresponding control cells (Panc28-Ctrl) orthotopically into the pancreas of nude mice. As shown in Figure 7, 5 of 5 mice in the control group (Panc28-Ctrl) developed big tumors, while only 2 of 5 mice in the Sema3E-knockout group (Panc28-S3EKO) developed tumors. Hence, knockout of Sema3E decreased rate of tumor incidence. In addition, tumor volume in the Panc28-S3EKO group was significantly lower than that of the Panc28-Ctrl group, indicating that knockout of Sema3E decreased tumor growth *in vivo*.

### Increased cell growth and migration via Sema3E overexpression is in part induced by the MAPK/ERK pathway

**Figure 8:**
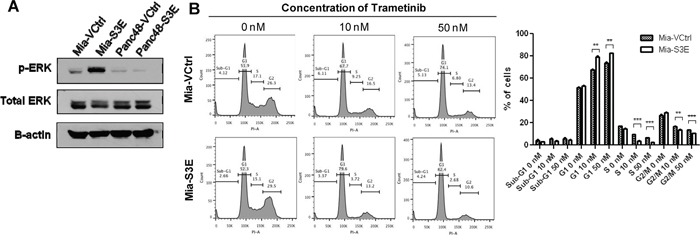
The activation of MAPK/ERK pathway contributes to cell proliferation in Sema3E-overexpressing MiaPaCa-2 cells **A**. Immunoblot analysis of Mia-S3E and Panc48-S3E cells and their respective controls shows that phosphorylated-ERK (pERK) levels were higher in Mia-S3E cells compared to Mia-VCtrl, while pERK levels in Panc48 cells remained the same regardless of Sema3E overexpression. **B**. Cell cycle analysis upon treatment of MiaPaCa-2 cells with MEK inhibitor trametinib shows that trametinib inhibited cell proliferation to a greater extent in Mia-S3E cells compared to Mia-VCtrl cells. This is reflected by the higher number of cells in G1 phase in Mia-S3E cells compared to Mia-VCtrl upon addition of trametinib, as well as fewer cells in both S phase and G2/M phase in Mia-S3E cells compared to Mia-VCtrl upon addition of trametinib (Student's *t-test*, **p<0.01, ***p<0.001). All data are represented as mean ± S.E.M, and are representative of at least 3 independent experiments.

To delineate the cellular pathways and mechanisms behind the observations we have made of Sema3E's effects on cell growth and migration, we performed a reverse phase protein array (RPPA) analysis on Mia-S3E vs. Mia-VCtrl cells. The results of the analysis are presented in Supplementary Table S1, which is a list of the most upregulated (>2-fold) and downregulated (<0.5-fold) proteins in Sema3E-overexpressing vs. control MiaPaCa-2 cells. The most significantly altered protein was phosphorylated-Erk1/2 (pERK1/2), which was upregulated almost 9-fold in Sema3E-overexpressing cells vs. the control. Immunoblot analysis (Figure 8A) confirmed the RPPA results, showing increased ERK1/2 phosphorylation in Mia-S3E cells compared to the control; however, pERK1/2 expression was generally low in Panc-48 cells, and there was also no difference in pERK levels in Panc48-S3E vs. Panc48-VCtrl cells, indicating that the ERK pathway is unlikely to be a major pathway regulating the proliferation of Panc-48 cells.

To determine if the MAPK/ERK pathway was involved in Sema3E's regulation of cell proliferation in MiaPaCa-2 cells, we used the MEK1/MEK2 inhibitor drug, trametinib, on Mia-VCtrl and Mia-S3E cells. The cells were treated with 0, 10, or 50 nM trametinib for 24 h, and were then harvested for cell cycle analysis. As shown in Figure 8B, treatment with 10 or 50 nM trametinib suppressed cell proliferation to a greater extent in Mia-S3E compared to Mia-VCtrl cells. This is indicated by a significantly greater number of cells in the G1 phase in Mia-S3E compared to Mia-VCtrl upon 10 or 50 nM trametinib treatment, as well as significantly fewer cells in S and G2/M phases in Mia-S3E compared to Mia-VCtrl upon 10 or 50 nM trametinib treatment, indicating that Sema3E may stimulate cell proliferation partly through MAPK/ERK pathway.

## DISCUSSION

Sema3E has been reported to be involved in several different cancers in the past few years, but our study is the first to report the functions of Sema3E in pancreatic cancer. By analyzing Sema3E expression in three separate sources of patient samples, we found that Sema3E was overexpressed in pancreatic cancer, and that patient samples expressing high levels of Sema3E were associated with poor survival. In addition, we found that Sema3E, despite being described and characterized as a secreted protein, was expressed in the nucleus of pancreatic cancer cells and that a high N/C ratio of Sema3E expression was associated with poorly-differentiated tumors, aggressive cancer, and a lower survival rate. Overexpression of Sema3E which increased tumorigenic and metastatic potential, supported our tissue analysis, while knockout of Sema3E elicited the opposite effects. By profiling Sema3E in pancreatic cancer, we show the importance and significance of Sema3E in the pathogenesis of pancreatic cancer, and highlight its potential roles as a prognostic marker as well as a therapeutic target.

Our study is the first to report the localization of Sema3E in the nucleus, since all previous reports of Sema3E have been focused on its characteristics and functions as a secreted protein. There has also been no other report on the localization of any other semaphorin protein in the cell nucleus. We have performed an algorithm analysis of the amino acid sequence of full-length Sema3E protein using an online nuclear localization sequence (NLS) prediction tool (NLS Mapper) [16], and results revealed putative NLS sequences near the C-terminus (results not shown). A similar predicted NLS sequence was obtained using the NucPred tool [17] (results not shown). We are currently investigating the effects of mutating these predicted NLS sequences on the cellular localization of Sema3E.

Our discovery that Sema3E was strongly expressed in the nuclei of tumor cells in PDAC, but not in normal acinar cells or tumor cells of pancreatic neuroendocrine carcinoma, could point to Sema3E's potential role as a transcription factor. Results from RPPA analysis of Mia-S3E vs Mia-VCtrl cells indicate that there were several proteins whose levels were increased upon Sema3E overexpression, and this could possibly be triggered by Sema3E's direct activation of gene transcription. A notable example is EGFR; in highly proliferating cells, including cancer cells, it is translocated to the nucleus, where it can activate transcription of genes involved in cell proliferation. Although EGFR lacks a DNA-binding domain, it can associate with other proteins to activate transcription of genes [18–20]. In the same way, Sema3E may be able to activate gene transcription; future chromatin immunoprecipitation sequencing (ChIP-seq) analysis may shed some light on whether Sema3E has transcription factor activity.

A subject of particular interest is the observation that upon overexpression of Sema3E cDNA in MiaPaCa-2 and Panc-48 cells, the cleaved 61 kDa product was increased in the culture supernatant, as well as in the cytoplasmic fraction of the cell lysates, but not in the nuclear fraction. This is a subject of our current investigation. It is plausible that a mechanism may exist to regulate the levels of the 61 kDa isoform in the nucleus, preventing its accumulation in the nucleus, considering the possibility that Sema3E could act as a transcription factor in the nucleus. A variety of mechanisms that regulate the nuclear accumulation of transcription factors have been described before. A few notable examples are: for Stat1, non-specific binding of DNA to the transcription factor protects it from dephosphorylation, retaining it in the nucleus [21]; for the GATA transcription factor GLN3, phosophorylation of the protein causes its binding to cytoplasmic protein URE2, preventing it from being transported to the nucleus [22].

Sema 3E expression differs markedly between cancer types. Upregulation of Sema3E in breast [4, 5, 8], colon [23], ovarian [7], and gastric cancer [24] was associated with progressively worse or more invasive/metastatic cancer. In contrast, Sema3E levels were found to be inversely associated with tumor progression in melanoma [25] and in another study of gastric cancer [26]. One explanation for the contrasting behavior of Sema3E between different types of cancers is the distinct tumor microenvironments of these disparate tumors. Sema3E also has multi-faceted effects on different cell types, dependent on factors such as the co-expression of Plexin D1 and Neuropilin-1 receptors [27]. Interestingly, Chen et al. [26] evaluated Sema3E expression in prostate, mammary, ovarian, and uterine cancer, and found, in agreement with previous reports [5–8, 23], that Sema3E was overexpressed in these cancers. Our observations of Sema3E overexpression in pancreatic cancer are similar to these reports. In addition, our study reveals the associated high Sema3E levels with worse survival in pancreatic cancer. A very recent study on gastric cancer has found that high Sema3E mRNA levels correlated with worse survival in human gastric cancer.

In an orthotopic xenograft mouse model generated by the implantation of Mia-S3E and Mia-VCtrl cells into nude mice, we found that Sema3E-overexpressing cells had a higher rate of tumor formation, and also formed bigger tumors compared to Mia-VCtrl. Conversely, orthotopic implantation of Sema3E-knockout and control Panc-28 cells into nude mice generated the opposite results. However, because of the less invasive nature of MiaPaCa-2 cells, we were unable to observe any metastasis of tumor cells to distant organs. We also showed that Mia-S3E tumors were more vascularized than Mia-VCtrl tumors, indicating that Sema3E overexpression may promote tumor angiogenesis, which in turn promotes greater tumor growth. This suggests that in an *in vivo* setting, Sema3E may also have paracrine effects on endothelial cell growth in the tumor microenvironment. This observation is in agreement with Christensen et al's study, which has reported that Sema3E, particularly the p61 kDa form, promoted endothelial cell migration via activation of ERK1/2 [5]. However, other reports have made contrasting observations, which are that both full-length and cleaved 61 kDa forms of Sema3E inhibited tumor angiogenesis, although they did not inhibit metastasis [6, 28]. One explanation for the increased vascularization we have observed could be that Sema3E may delineate the growth of blood vessels through a gating mechanism, which has been described in axonal growth; the co-expression of Neuropilin-1 in addition to Plexin D1 on axonal projections induces attraction instead of repulsion, in response to Sema3E [29].

As to Sema3E's role on tumor growth, a few studies have reported, in contrast to our findings, that Sema3E inhibits tumor growth, sometimes regardless of its effects on cancer metastasis. Cassaza et al. [30] found that both the full-length uncleavable mutant form as well as the furin-cleaved p61 form of Sema3E inhibited tumor growth *in vivo* (but not *in vitro*), presumably via Sema3E's paracrine effects on inhibiting tumor-induced angiogenesis. The same inhibitory effects on tumor growth were observed in a study of the overexpression of full-length uncleavable mutant Sema3E in glioblastoma cell lines, *in vitro* and *in vivo* [28]. Sema3E overexpression in gastric cancer cell lines yielded the same result – reduced cell proliferation *in vitro* due to less G1 to S phase transition as well as promotion of apoptosis. Importantly, although furin was expressed in these cell lines, the authors could not detect the p61 kDa isoform of Sema3E via immunoblot analysis, and concluded that the anti-tumor effects observed were induced by the full-length form. In our study, upon overexpression of wild-type Sema3E (via lentiviral transduction of Sema3E cDNA clone), we could detect an increase in both full-length and cleaved 61 kDa Sema3E. Given that the 61 kDa Sema3E protein was increased in our overexpression cell lines, the effects we see, i.e. increased cell proliferation and greater tumor growth, could possibly be caused by the 61 kDa protein, although it is unclear if the increase in the full-length protein contributed to these effects too.

As for Sema3E's role in cancer metastasis, some studies have reported Sema3E to promote cell migration and invasiveness *in vitro* and metastasis *in vivo* [6, 30], and these studies have also found that the p61 kDa isoform of the protein was primarily responsible for these effects, either through transactivating ErbB2/Neu kinase signaling, or by inducing nuclear localization of Snail1 via the activation of PI3K and MAPK pathways [7]. While other studies have reported Sema3E to have inhibitory effects on melanoma and prostate cancer metastasis [25, 31], they failed to identify and discriminate between the full-length and the cleaved forms of the protein, making it difficult to discern between distinct differences between types of cancer or Sema3E cleavage. For our study, we found that overexpression of both full-length and cleaved Sema3E in Panc-48 cells could promote cancer cell migration and invasion, while knockout of full-length Sema3E in Panc-28 cells inhibited cell migration. Our results, in part, corroborate the conclusions reached by the above-mentioned studies. It is possible that full-length Sema3E could promote cell migration/invasion via yet unidentified pathways. Results from our RPPA analysis (Supplementary Table S1) have revealed several interesting proteins associated with metastasis, in particular fibroblast-specific protein (FSP1), also known as S100A4 [32].

The MAPK/ERK pathway is widely known to be involved in cancer cell proliferation, differentiation, apoptosis, and migration [33]. Sema3E has also been shown to activate this pathway in neuroblastic cells [34], as well as in cancer cells [6–7]. MEK kinase is directly upstream of ERK1/2, and inhibition of MEK with trametinib blocks the activation of downstream effector molecules, including the phosphorylation of ERK1/2. As such, trametinib has been shown to be a potent inhibitor of cancer cell proliferation [35] and has been approved by the FDA for the treatment of melanoma with BRAF mutations. In our study, we found that overexpression of Sema3E induced greater phosphorylation of ERK1/2 in MiaPaCa-2 cells, and that treatment of MiaPaCa-2 cells with trametinib suppressed cell proliferation to a greater extent in Sema3E-overexpressing cells, thereby suggesting that the MAPK/ERK pathway could be one of the mechanisms involved in the proliferation of Sema3E-overexpressing pancreatic cancer cells.

In conclusion, we have shown that Sema3E is overexpressed in human pancreatic cancer samples, and that high levels of Sema3E expression is associated with poor survival. In addition, we have shown that high N/C ratio of Sema3E expression is associated with more aggressive disease progression as well as worse survival. Particularly, we made the discovery that Sema3E, a known secreted protein, was also localized in the cell nucleus. Collectively, we have established the significance of Sema3E protein in the clinical setting, as well as identified its tumorigenic functions on PDAC cells *in vitro* and *in vivo*. Our findings point towards the potential use of Sema3E as a prognostic marker for predicting pancreatic cancer survival, as well as the potential targeting of Sema3E or its signaling pathways as a novel treatment for pancreatic cancer.

## MATERIALS AND METHODS

### Cells, antibodies (Abs)

Human pancreatic cancer cell lines, BxPC-3, Panc-1 and MIA PaCa-2 were purchased from the American Type Culture Collection (ATCC, Rockville, MD). Other human pancreatic cancer cell lines, Panc-28 and Panc-48, were obtained from Dr. Craig Logsdon at MD Anderson Cancer Center (Houston, TX). Sequencing data obtained from a study by Jones et al. [10] on pancreatic cancer cell lines did not uncover any mutations in *SEMA3E*. According to the Broad-Novartis Cancer Cell Line Encyclopedia (CCLE), mutations were also not found in the *SEMA3E* gene of the MiaPaCa-2 cell line. The human pancreatic ductal epithelium (HPDE) cells given generously by Dr. Ming-Sound Tsao (Division of Applied Molecular Oncology, Ontario Cancer Institute, Toronto, Ontario, Canada). HPDE cells were cultured in keratinocyte serum-free medium (SFM) (Thermo Fisher Scientific, Grand Island, NY) supplemented with 5 ng/ml of recombinant epidermal growth factor (EGF) and 50 μg/ml bovine pituary extract (BPE). All other pancreatic cancer cell lines were cultured in Dulbecco Modified Eagle's Medium (DMEM) (Lonza Houston, Houston, TX) supplemented with 10% fetal bovine serum (FBS) and 4 mM L-glutamine. Rabbit anti-human Sema3E polyclonal Ab (used for immunohistochemistry), and rabbit anti-human/mouse Lamin A Ab (used for immunoblot analysis) were acquired from Abcam (Cambridge, MA). Goat anti-human/mouse Sema3E polyclonal Ab (used for immunoblot analysis) was acquired from R&D Systems (Minneapolis, MN). Mouse anti-human/mouse β-actin monoclonal Ab (used for immunoblot analysis) was acquired from Sigma (St. Louis, MO). Mouse anti-human/mouse GAPDH monoclonal Ab (used for immunoblot analysis) was acquired from Santa Cruz Biotechnology (Dallas, TX).

### Human tissue samples

Tissue microarray samples were retrieved from archives at Department of Pathology, Michael E. DeBakey VA Medical Center according to an approved human IRB protocol by Baylor College of Medicine. Detailed demographic information was obtained from patients' records including age, gender and survival. Clinical stage was also recorded based on the American Joint Commission on Cancer staging system. Tumor histologic types included: adenocarcinoma (25 cases), neuroendocrine carcinoma (7 cases), sarcomatoid carcinoma (1 case), neuroendocrine tumor (6 cases), intraductal papillary mucinous neoplasm (2 cases), serous microcystic adenoma (2 cases) and pancreatic intraepithelial neoplasia (1 case). Hematoxylin & Eosin stained sections were reviewed by a surgical pathologist (S. Lai) to confirm the original histopathological diagnosis. Duplicate 1-mm cores from two representative formalin-fixed, paraffin-embedded blocks of each patient were used to generate a tissue microarray (TMA).

### Immunohistochemical analysis of human tissue samples

A 5-μm section was cut from TMA, deparaffinized in Bond Dewax Solution (Leica Biosystems, Newcastle, United Kingdom) and rehydrogenized in descending grades (100-70%) of ethanol. Antigen recovery was achieved by 20 minutes heat-induced epitope retrieval. Immunohistochemical (IHC) stains were performed using an automated tissue-staining system (Bond Polymer Refine Detection System-Leica Biosystems, Newcastle, United Kingdom). Tissue sections were stained with polyclonal rabbit anti-Sema3E antibody following manufacturer's instructions. Bound Sema3E antibody was detected using polymer reagent conjugated with horseradish peroxidase and affinity purified goat-anti-rabbit antibody followed by the 3,3′-Diaminobenzidine (DAB) Chromogen Kit (Leica Biosystems, Newcastle, United Kingdom). Appropriate positive and negative controls were performed. Each IHC slide was evaluated by a surgical pathologist (S. Lai) who was blinded to patient outcome. The stains with none, weak, moderate or strong intensities in nuclear or cytoplasmic of the cells were manually scored as 0, 1+, 2+ and 3+, respectively. A final score was obtained by the sum of multiplying both intensity and extension values [15]. The scores ranged from 0 to 300. The nuclear-to-cytoplasm ratio (N/C ratio) of Sema3E expression was computed by taking the score of Sema3E staining in the nucleus divided by the score of Sema3E staining in the cytoplasm. Log_2_(N/C ratio) was computed by taking the logarithm of the N/C ratio to base 2.

### Real-time PCR (RT-PCR) analysis

Total RNA was extracted from pancreatic cancer cells using the RNAqueous total RNA isolation kit (Life Technologies, Grand Island, NY), according to manufacturer's instructions. Briefly, cells were first lysed with lysis buffer, and an equal volume of 64% ethanol was added. The mixture was passed through a mini-column via centrifugation at 10,000 x g, and washed with buffer 1 and buffer 2/3. RNA was eluted by adding 50 μl of elution buffer onto the column and centrifugation at 10,000 x g. To generate cDNA, the eluted RNA was reverse transcribed using the iScript cDNA synthesis kit (Bio-Rad, Hercules, CA). Sema3E mRNA expression was analyzed via RT-PCR using the iCycler iQ system (Bio-Rad). The following primer sequences for detecting Sema3E were used: 5′-CTGTTTCACCTGGAATCACCC-3′ (sense) and 5′-GTGCGGATATGGGCCAGTC-3′ (antisense). For detecting levels of the internal control GAPDH, these primer sequences were used: 5′-ATGACAACTTTGGTATCGTGGA-3′ (sense) and 5′-GTAGAGGCAGGGATGATGTTCT-3′ (antisense). RT-PCR was performed using the SYBR green PCR supermixes (Bio-Rad). The PCR reaction consisted of: 100 nM of each primer, 1 μl cDNA template, and 19 μl of the supermix, and the reaction was ran for 40 cycles of 95°C for 20 seconds and 60°C for 1 minute. Each sample was run in triplicate to detect levels of both Sema3E and GAPDH. Relative Sema3E levels were normalized to glyceraldehyde-3-phosphate dehydrogenase (GAPDH). The relative mRNA level was presented as 2^(*threshold cycle* for GAPDH − *threshold cycle* for *gene of interest*)^, and fold-change in expression of Sema3E was calculated as the relative mRNA level of Sema3E in a given condition vs. the control condition.

### Immunoblot analysis

To lyse cells, cell pellets were resuspended in cold lysis buffer (RIPA buffer (Sigma) with addition of a protease inhibitor cocktail (Sigma) and left on ice for 30 min. Lysates were then centrifuged at 14,000 x g for 15 min at 4°C, and the supernatant was acquired for subsequent immunoblot analysis. For protein fractionation into cytoplasmic and nuclear fractions, the Subcellular Protein Fractionation Kit for Cultured Cells (Thermo Fisher) was used, according to manufacturer's instructions. Protein concentrations of lysate supernatants were determined using the Pierce BCA Protein Assay kit (Life Technologies), according to the manufacturer's instructions. 30 μg of total cellular protein was separated with 10% SDS-polyacrylamide gel electrophoresis and then transblotted at 4°C at 80 volts for 90 min, onto a 0.2 μm nitrocellulose membrane (Bio-Rad, Hercules, CA). The membrane was blocked with 5% milk; 0.1% Tween-20-TBS buffer for 2 h at 4°C, probed with goat anti-Sema3E (1:1000 dilution) or mouse anti-β-actin (1:4000 dilution) or mouse anti-GAPDH or rabbit anti-Lamin A at 4°C overnight, and washed with 0.1% Tween-20-TBS buffer, before being incubated with a horse peroxidase-linked secondary antibody (1:2000-4000 dilution) for 1 h at room temperature. The membrane was washed again with 0.1% Tween-20-TBS buffer, and immunoreactive bands were then detected using an enhanced chemiluminescent (ECL) plus reagent kit (Thermo Fisher).

### Immunocytochemistry

Cells were seeded at subconfluency in Lab-Tek II chamber slides (Thermo Fisher) and left to adhere for at least 6 h. Cells were fixed with 4% paraformaldehyde in PBS and permeabilized with PBS containing 0.2% triton, and incubated with 3% hydrogen peroxide in PBS to inactivate endogenous peroxidase activity. Cells were then blocked with 2% BSA in PBS for 1 h, then incubated with goat anti-Sema3E antibody (1:200 dilution) in the blocking buffer overnight at 4°C. Cells were then incubated with anti-goat-HRP secondary antibody (1:400 dilution), and developed with DAB substrate solution. A haematoxylin counterstain was used, and slides were dehydrated and mounted with xylene-based mounting medium. The slides were visualized and imaged using the Olympus RX41 upright microscope (Olympus Life Science, Center Valley, PA) equipped with a Leica DFC300 FX camera (Leica Biosystems).

### Generation of Sema3E-overexpressing stable cell lines

MiaPaCa-2 and Panc-48 cells were used to generate Sema3E-overexpressing cell lines. In both Jones et al [10] and Biankin et al. [9] studies, *SEMA3E* gene changes were reported to be mostly copy number variations (CNV). While 18 samples had significant increased copy numbers in SEMA3E gene, only 1 sample contained a (non-silent) mutation [9]. Therefore, it is more clinically relevant to study the effects of wildtype Sema3E overexpression in pancreatic cancer. The wildtype Sema3E cDNA-expressing lentiviral-capable plasmid was a kind gift from Luca Tamagnone and was generated as reported previously [5]. This plasmid and a control plasmid was co-transfected along with lentivirus-generating plasmids into 293T cells. Culture supernatants containing Sema3E-expressing or control-expressing lentivirus were collected, passed through a 0.45 μm filter, and added to the target cells, as previously described [36], which ensured high transduction efficiency without a need to select individual cell clones. The overexpression of Sema3E in each cell line was confirmed by immunoblot and RT-PCR analyses.

### Generation of stable Sema3E-knockout cells

CRISPR/Cas9 system was used to generate Sema3E knockout PC cell lines, sgRNA/Cas9 all-in-one expression plasmid targeting to SEMA3E (NM_001178129.1) including pCRESPR-CG01 a,b,c and scrambled plasmid DNA were purchased from Genecopia (Rockville, MD). About 3 × 10^5^ Panc-28 cells were plated in each well on a 6-well plate. After 24 h, the cells were transfected with 1 μg of sgRNA/Cas9 all-in-one expression plasmid targeting SEMA3E a.b.c sites or control plasmid following the manufacturer's instructions. At 24 h post transfection, cells were split at 1:10 ratio into a 10 cm dish containing DMEM medium with 2 mg/ml G418, the antibiotic used for selection of positive clones. After growing for 3 additional days, cells were split and cultured again for 3 weeks. mCherry-positive cell clones were picked and amplified. Protein and genome DNA were extracted. The knockout of Sema3E was confirmed by Western blot and DNA sequencing. Sequencing primers used were:

semcrisL 5′-GGGGTGGGTAGTATCAGCTG-3′ and

semcrisR 5′-CAGCCTGACAAATGGCACTT-3′, covering target sites a and b

semcrisLc 5′-TCAGTGATTCAGTTCTTTGT GGT-3′ and

semcrisRc 5′-AAAGCCCAGCGATACGTTTC-3′, covering target site c.

### Cell proliferation assay

For the MTT cell viability assay, cells to be analyzed were seeded at 5000 cells/well in a 96 well plate, and serum-starved with media containing 0% FBS for 24 to 36 h (depending on doubling time of cell line), followed by incubation with DMEM media containing 1% FBS. Cell growth was assessed at days 0, 2, 4 and 6 upon release from serum starvation. To assess cell growth at each time point, 10 μl of 5% (3-(4,5-Dimethylthiazol-2-yl)-2,5-Diphenyltetrazolium Bromide) (MTT) in 100 μl media was added to the cells, and incubated for 4 h at 37°C. Media was removed from the cells, and 100 μl of DMSO was added to solubilize the purple formazan crystals formed. Absorbance was read at 495 nm using the EL-800 universal microplate reader (Bio-Tek Instruments, Winooski, VT).

### Colony formation assay

The colony formation assay is a measure of the clonogenic, or tumorigenic potential of cells based on the ability of individual cells to form colonies via unlimited cell division [37]. Cells were seeded at a density of 500 cells/well in complete DMEM media in a 6-well plate, and incubated at 37°C for 14 days to allow for the formation of colonies. The culture medium was replaced every 3 to 4 days. At day 14, colonies were fixed with glutaraldehyde (6.0% v/v), stained with crystal violet (0.5% w/v), and counted using the ImageJ software.

### Cell cycle analysis

Cells were serum-starved for 36 to 48 h (depending on doubling time of cell line) followed by the addition of media containing 2% serum and collected after 4 or 8 h. Cells were harvested and fixed in ice-cold 70% ethanol for at least 1 h at 4°C, washed with PBS, and incubated with a solution containing 50 μg/ml propidum iodide, 10 μg/ml RNase, 0.05% triton-X 100 for 30 min at 37°C, or 2 h at room temperature (25°C). Cells were then immediately analyzed on a flow cytometer using the 488 nm laser (LSRII, BD Biosciences, San Jose, CA). Analysis of the data obtained on the flow cytometer was performed using the FlowJo software (FlowJo, Ashland, OR).

### Wound healing migration assay

Cell migration *in vitro* is an indication of the invasive and metastatic potential of a cancer cell, and in a wound healing assay, the movement of cells towards the wound area in a serum-starved setting measures the rate of cell migration *in vitro*. Cells were cultured to full confluency in 6-well plates, and serum-starved with media containing 0% FBS for at least 24 h before scratches were created, and maintained with media containing 0% FBS for the entire duration of the experiment. Scratches, or wounds, were created by drawing a straight line across the cells with a sterile 200 μl pipette tip. Wound healing was observed at 0, 24, and 72 h at the scratch line, and representative fields were photographed using an inverted phase contrast microscope equipped with a digital camera. The wound area at the various timepoints was quantified using the MRI Wound Healing Tool (http://dev.mri.cnrs.fr/projects/imagej-macros/wiki/Wound_Healing_Tool), a macro toolset designed for use with the ImageJ software.

### Transwell migration and invasion assays

Cell migration or invasion was determined using the Boyden chamber assay. Cell migration was performed using uncoated transwell chambers, while cell invasion was performed using transwell chambers coated with matrigel matrix (Corning, Fisher Scientific, Waltham, MA). Briefly, 200 μl of cells were added at 5 × 10^5^ cells/ml to the upper compartment of a transwell chamber with a 0.8 μm pore size (Corning). Complete DMEM media was added to the well which the chamber is inserted into. After incubation at 37°C for 24 h (for Panc-48) or 48 h (for MiaPaCa-2), cells were labeled with calcein-AM (Molecular Probes, Eugene, OR) and fixed with 4% paraformaldehyde in PBS. The total amount of cells in the upper chamber as well as cells that had migrated through the membrane was first quantified by reading the fluorescence of calcein AM-labeled cells at an excitation wavelength of 485 nm and an emission wavelength of 520 nm using a microplate reader (Bio-Tek, Winooski, VT), and then cells in the upper chamber were removed via scraping with a cotton tip and washing with PBS, and the amount of cells left on the bottom surface of the membrane, the cells that had migrated, were quantified again by reading fluorescence at the same wavelengths. Migration rate was presented as: fluorescence reading before scraping (total cells)/fluorescence reading after scraping (migrated cells) x 100%.

### Orthotopic pancreatic cancer mouse model

MiaPaCa2 or Panc-48 cell lines with stable Sema3E overexpression and corresponding vector control or Panc-28 cell lines with stable Sema3E knockout and corresponding vector control were cultured to subconfluency, then harvested and resuspended in sterile PBS for orthotopic implantation into 6 week old athymic female nude mice (Charles River Laboratories, Wilmington, MA). 2 × 10^6^ cells were inoculated directly into the pancreas as previously described [38]. For intrapancreatic injection, mice were anesthetized with isoflurane gas, and a 0.5 cm incision was made in the left subcostal region. The spleen was pulled out slowly and carefully to expose the pancreas. Tumor cells were injected in a volume of 50 μL into the body of the pancreas. The pancreas and spleen were carefully placed back into the body cavity, and the incision site was closed with sutures. After implantation of the tumor cells, mice were examined regularly for signs of weakness and disease. Mice were euthanized at 8 weeks post-implantation (or earlier when the animal reaches a moribund state) using isoflurane gas anesthesia followed by cervical dislocation. The euthanized mice were then evaluated macroscopically for the presence of pancreatic tumor and metastases, as well as other indicators of disease, such as a jaundiced liver or an enlarged spleen. Tumors (if any) were explanted from the pancreas of the mice. Tumor size was measured using digital calipers and tumor volume was determined with the following formulas: tumor volume (cm^3^) = [length (cm)] × [width (cm)]^2^ × 0.52. Part of the tumor tissue and organs of potential metastasis were fixed and embedded in paraffin for histological analysis.

### Immunohistochemical analysis of mouse tissues

Fixed tissues were processed using the Tissue-Tek VIP 6 Vacuum Infiltration Processor (Sakura, Alphen aan den Rijn, The Netherlands). Processed tissues were embedded in paraffin into tissue blocks using the Tissue-Tek TEC Tissue Embedding Console System (Sakura). Blocks were cut into 5 μm thick sections and mounted onto glass slides. Tissue sections were dipped in xylenes, followed by a series of decreasing concentrations of alcohol to rehydrate the sections. Endogenous peroxidase activity was blocked via incubation of the sections with 3% hydrogen peroxide in methanol. Antigen retrieval was performed by incubating the sections in pH 6 citrate buffer in a pressure cooker for 15 min, and sections were left to cool in the buffer. Sections were blocked for 1 h at room temperature with 2% BSA in PBS containing 0.02% triton. For Ki-67 staining, sections were incubated with mouse anti-human Ki-67, clone MIB-1 (Dako, Carpinteria, CA) diluted 1:100 in blocking buffer overnight at 4°C, and then incubated in anti-mouse-HRP secondary antibody diluted 1:400 in blocking buffer for 1 h at room temperature. For CD31 staining, sections were incubated with rat anti-mouse CD31, clone MEC13.3 (BD Biosciences), diluted 1:40 in blocking buffer for 3 h at room temperature, then incubated with a broad spectrum biotinylated secondary antibody from the Histostain-Plus IHC kit (Thermo Fisher), followed by incubation with streptavidin-HRP from the same kit, following the manufacturer's instructions. Sections were developed using the DAB plus Chromogen Solution (Dako), and counterstained with haematoxylin. Sections were dehydrated, and mounted with a xylene-based mounting medium. Slides were visualized and imaged using the Olympus RX41 upright microscope (Olympus Life Science, Center Valley, PA) equipped with a Leica DFC300 FX camera (Leica Biosystems). The extent of Ki-67 expression in tumor tissues was quantified using the ImmunoRatio application developed by Tuominen et al. [39] plugged into ImageJ, which determines the number of DAB-stained nuclei proportional to the total number of nuclei in the image.

### Reverse phase protein array

MiaPaCa-2 control and Sema3E-overexpressing cells were lysed in a procedure similar to the immunoblot assay. Protein lysates were sent to the Baylor College of Medicine Reverse Phase Protein Array (RPPA) Core Facility for high-throughput RPPA analysis. A total of 204 proteins were analyzed using validated antibodies to these proteins. RPPA analysis was carried out in a procedure previously described by Dong et al. [40]. In brief, protein lysates were denatured in SDS buffer, and then loaded into 384-wellplates. Samples were then printed onto slides using the Aushon 2470 Arrayer (Aushon Biosystems, Billerica, MA). Slides were incubated with the various antibodies and corresponding secondary antibodies using the Dako Autostainer (Dako). Negative control slides were prepared by incubating slides with the same reagents excluding the primary antibody. Total protein levels were determined using the Sypro Ruby Protein Stain (Molecular Probes, Eugene, OR). Stained slides were scanned using the GenePix AL4200 scanner (Molecular Devices, Sunnyvale, CA), and images were analyzed with GenePix Pro 6.0 (Molecular Devices). Fluorescence signal of each spot, of each antibody, was subtracted from the background signal, and normalized using a group-based normalization method as described by Grubb et al. [41]. Significant change of protein levels between the samples were determined by a fold-change of at least 1.5.

### Treatment of cells with MEK1/2 inhibitor trametinib

To determine the effects of MEK inhibitor drug Trametinib (ApexBio, Houston, TX) on MiaPaCa-2 cells, a concentrated stock solution of Trametinib was first weighed and dissolved at 100 mM in DMSO. The stock solution was diluted in DMEM media and added to cells at 10 or 50 nM for 24 h, before any analysis was made.

### Statistical analyses

The Student's paired *t-test* was used for comparison between tumor and normal matched control tissues in the GEO GDS4103 dataset [42]. The log-rank test was performed for comparing survival curves between low and high Sema3E-expressing patient populations from TCGA PAAD RNAseq exon expression data [43, 44]. The non-parametric Wilcoxon signed-rank test was used for comparison between poorly- to moderately-differentiated and well-differentiated tumors. The non-parametric Kruskal-Wallis test by ranks was used for comparison of more than two populations/groups. Two-way ANOVA analysis was used for the MTT assay and wound healing assay quantification. All other statistical analyses were performed using the Student's unpaired *t-test*. P values <0.05 were considered significant.

## SUPPLEMENTARY FIGURES AND TABLE


