# Supplementary material for: Overexpression of Semaphorin-3E enhances pancreatic cancer cell growth and associates with poor patient survival
Source: Oncotarget. 2016 Nov 29;7(52):87431–48. doi: 10.18632/oncotarget.13704 (PMC5349999; doi:10.18632/oncotarget.13704)
Supplement: Supplementary file 1 [file oncotarget-07-87431-s001.pdf]

Overexpression of Semaphorin-3E enhances pancreatic cancer cell growth and associates with poor patient survival

SUPPLEMENTARY FIGURES AND TABLE

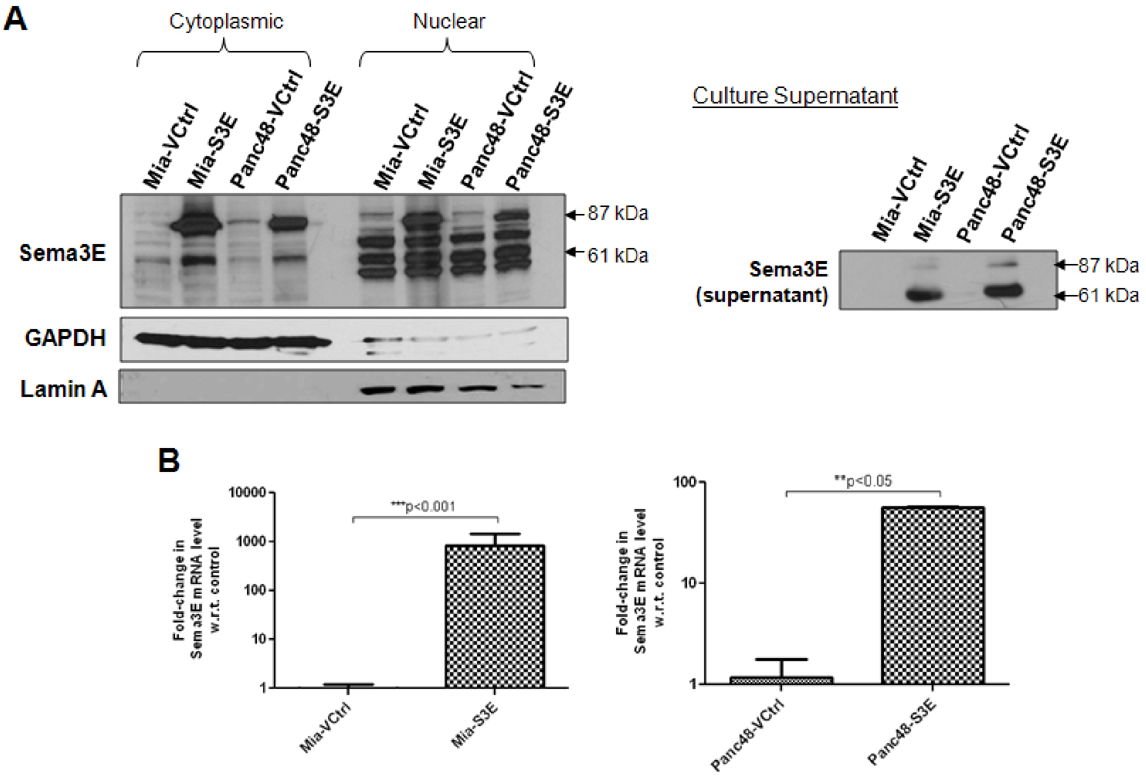

Supplementary Figure S1: Sema3E expression is increased in both the nucleus and cytoplasm in Sema3E-overexpressing cell lines. A. Subcellular fractionation was performed on vector control-transduced cells (Mia-VCtrl and Panc48-VCtrl) and Sema3E cDNA-transduced cells (Mia-S3E and Panc48-S3E). Immunoblot analysis shows that in the cytoplasmic fractions (left panel), there was a significant increase in Sema3E protein expression, in both the full-length 87 kDa protein and furin-cleaved 61 kDa protein fragment, in Mia-S3E and Panc48-S3E cells compared to their respective controls. In the nuclear fractions (left panel), only the full-length protein was increased in the Sema3E-overexpressing cells compared to the controls. Two other unidentified/unknown bands, in addition to the 61 kDa and 87 kDa bands, were observed. Both the 61 kDa and full-length 87 kDa protein were also present in the culture supernatant of Mia-S3E and Panc48-S3E cells but not in the controls (right panel). **B.** RT-PCR analysis shows a significant fold-increase in Sema3E gene expression in Mia-S3E (Student's t-test, ***p<0.001) and Panc48-S3E cells (Student's t-test, **p<0.01) compared to their respective controls.

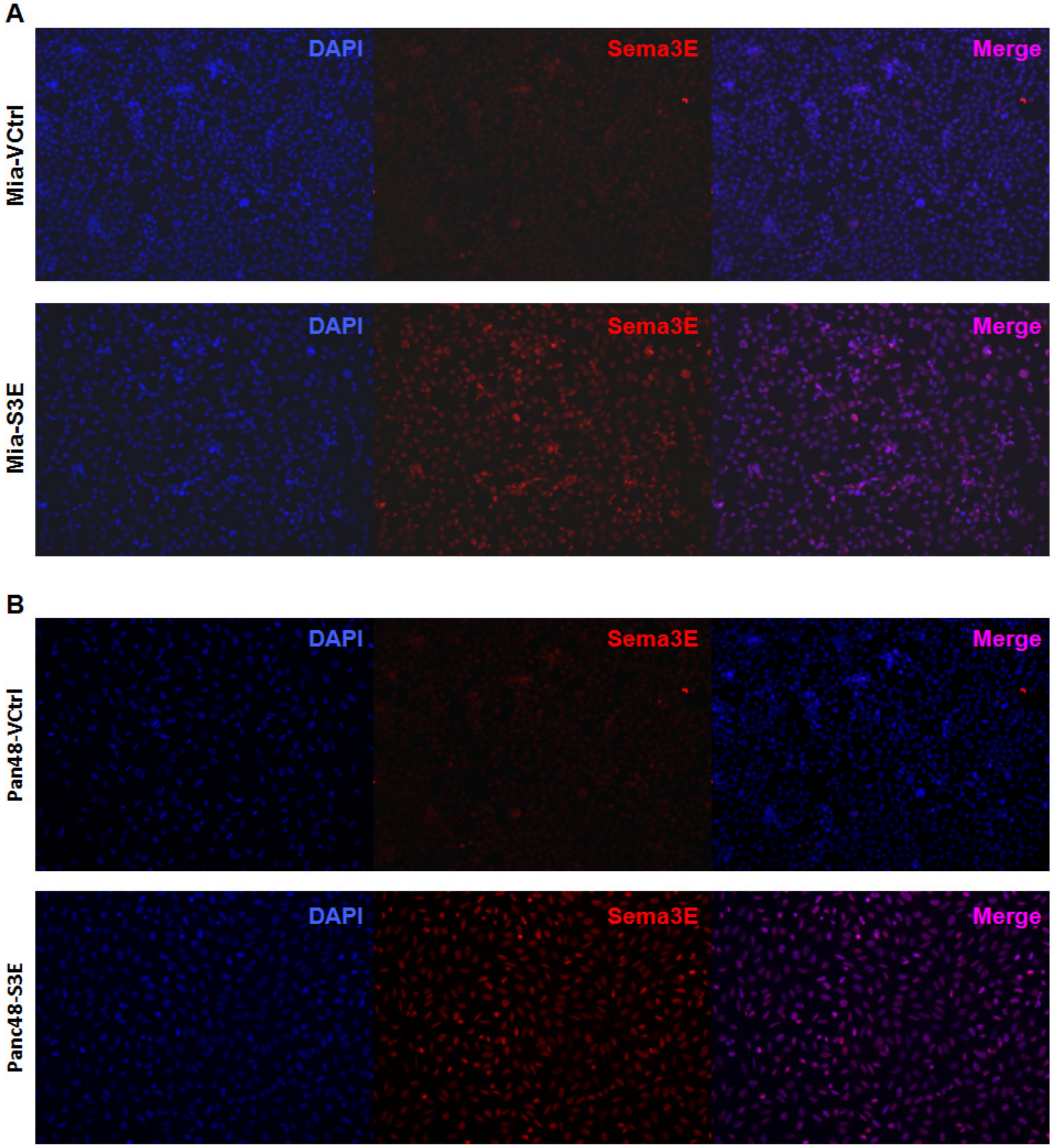

Supplementary Figure S2: Immunofluorescence staining of Sema3E expression in Sema3E overexpressed cells. Mia-S3E and Panc48-S3E cells and their controls shows that there is a significant increase in Sema3E protein expression in S3E-overexpressing cells compared to the controls in both the cytoplasm and the nucleus, but especially in the nucleus. Blue color is for DAPI staining in the nucleus, red color is for Sema3E staining. After merging the two pictures, purple color is the indication of Sema3E localization mainly in the nucleus.

Supplementary Table S1: Significantly up- and down-regulated proteins in Mia-S3E vs. Mia-VCtrl cells as identified by RPPA analysis

Protein	Fold Change (Mia-S3E vs. Mia-VCtrl)
p-p44/42MAPK(Erk1/2)(T202/Y204)	8.909141207
FSP1/S100A4	2.971429258
KLF4	2.814975424
Integrinb4	2.81052884
p21	2.692373231
LRP6	2.566232434
p27/KIP1(C-term)	2.017179339
p-Stat3(S727)	1.866475526
Vimentin(D21H3)	1.822892956
PI3Kp85	1.813637807
MEK6	1.797559966
p-MEK1/2(S217/221)	1.751305479
Bad	1.696594105
p-Bad(S112)	1.675391666
AMPKa(F6)	1.640102627
LDHA	1.61980421
Stat3(D3Z2G)	1.605838639
FoxO1(C29H4)	1.594812326
p-Akt(S473)	1.539911207
SOX9	1.513502772
p-p70S6K(T412)	1.502199291
p-FAK(Y397)	0.63197919
p-SHC(2431)(Y317)	0.588992037
Beta-Catenin	0.460818082
